# Phytochemical Analysis of Moroccan *Mercurialis annua* L. and Its Nephroprotective Activity: Evidence Supporting Traditional Renal Uses

**DOI:** 10.1002/cbdv.71802

**Published:** 2026-09-30

**Authors:** Abdeljalil Elmahdaoui, Manal Zefzoufi, Lansine Diakité, Mohammed Haida, Rabiaa Fdil, Aziz Auhmani, Stefania Garzoli, Zahra Sokar

**Affiliations:** ^1^ Laboratory of Pharmacology Neurobiology, Anthropology and Environment Department of Biology Faculty of Sciences Semlalia University Cadi Ayyad Marrakech Morocco; ^2^ Team of Bioorganic Chemistry Faculty of Sciences University Chouaïb Doukkali El Jadida Morocco; ^3^ Water, Biodiversity and Climate Change Laboratory Faculty of Sciences Semlalia Cadi Ayyad University Marrakesh Morocco; ^4^ Laboratory of Molecular Chemistry Unit of Organic Synthesis and Physico‐Molecular Chemistry Department of Chemistry Faculty of Sciences Semlalia University Cadi Ayyad Marrakech Morocco; ^5^ Department of Chemistry and Technologies of Drug Sapienza University Rome Italy

**Keywords:** acute toxicity, enzyme redox, *Mercurialis annua L*, nephroprotective, oxidative stress, UHPLC–ESI/MS

## Abstract

*Mercurialis annua L*. (Euphorbiaceae) is traditionally used to treat renal disorders, although its nephroprotective activity remains poorly documented. This study characterized the phytochemical profile of the Moroccan hydroethanolic extract by UHPLC–ESI/MS, evaluated its acute toxicity, and investigated its protective effect against cyclophosphamide‐induced nephrotoxicity in rats. UHPLC–ESI/MS tentatively identified phenolic and flavonoid constituents, including isorhamnetin derivatives, taxifolin, epicatechin, and hydro‐*p*‐coumaric acid hexoside. No mortality or treatment‐related toxicity was observed up to 5000 mg/kg. Oral administration of the extract (200 and 400 mg/kg) significantly reduced serum creatinine, blood urea nitrogen, kidney weight, and malondialdehyde levels while restoring superoxide dismutase and peroxidase activities. Histopathological examination confirmed marked attenuation of renal damage, particularly at 400 mg/kg. These findings provide the first phytochemical characterization of Moroccan *M. annua* and support its potential as a safe natural nephroprotective agent through antioxidant‐related mechanisms.

## Graphical abstract

*Mercurialis annua* L.: Phytochemistry and Nephroprotective Activity.

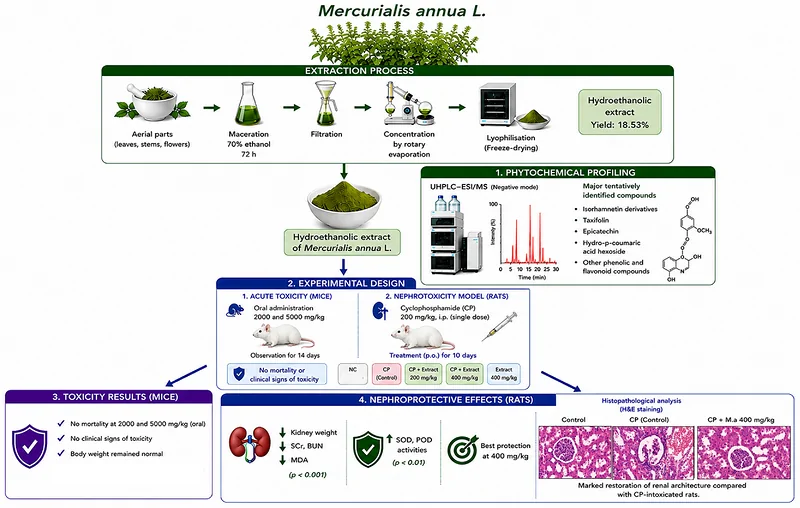

## 1 Introduction

Nephrotoxicity is a frequent adverse effect associated with a wide range of therapeutic agents, including anticancer drugs, antibiotics, and immunosuppressants [1]. The kidneys are highly vascularized and metabolically active and are particularly vulnerable to xenobiotic‐induced damage [2]. Oxidative stress, inflammation, and apoptosis are key mechanisms underlying drug‐induced renal injury, leading to impaired glomerular filtration, elevated serum biomarkers, and structural alterations such as tubular necrosis and glomerular atrophy [3]. The lack of effective protective therapies against nephrotoxicity remains a major clinical challenge [1].

Cyclophosphamide (CP) is a chemotherapeutic and immunosuppressive drug that is well known for its nephrotoxicity [4]. Cyclophosphamide metabolism leads to the formation of two cytotoxic compounds: phosphoramide mustard, responsible for the antineoplastic effect, and acrolein, a highly reactive compound with a short biological half‐life that is thought to be a major contributor to nephrotoxicity [4]. Acrolein induces intensive production of ROS, weakening the antioxidant system, and enhances inflammation in tissues, which could explain the potential toxicity of cyclophosphamide [4]. The resulting oxidative stress disrupts cellular redox balance, triggers inflammation, and damages renal tissues, leading to elevated serum urea and creatinine, lipid peroxidation, and histopathological alterations such as tubular degeneration and glomerular atrophy [5]. These pathological features closely resemble the mechanisms underlying drug‐induced acute kidney injury observed in patients receiving nephrotoxic medications, making cyclophosphamide a clinically relevant experimental model for evaluating nephroprotective interventions [6]. Although several experimental models, including cisplatin‐, gentamicin‐, adenine‐, and ischemia‐reperfusion‐induced nephrotoxicity, are available, each reproduces distinct mechanisms of renal injury [7]. In the present study, cyclophosphamide was selected as the experimental model because its nephrotoxicity is predominantly driven by oxidative stress and inflammation, two major mechanisms involved in drug‐induced renal injury [6]. Consequently, this model is particularly suitable for evaluating the nephroprotective potential of antioxidant‐rich medicinal plants and natural therapeutic strategies against oxidative stress‐mediated kidney damage [8].

Oxidative stress is a major contributor to cyclophosphamide‐induced nephrotoxicity; therefore, antioxidant‐based strategies have attracted considerable attention for preventing or attenuating renal injury [9]. Several medicinal plants rich in phenolic compounds have demonstrated promising nephroprotective effects in experimental CP‐induced renal injury. For example, extracts of *Moringa oleifera*, *Ocimum gratissimum*, *Croton macrostachyus*, and *Tinospora cordifolia* have been reported to reduce oxidative stress, improve renal biochemical markers, and preserve kidney histological architecture through their antioxidant and anti‐inflammatory activities [10, 11]. These studies support the concept that polyphenol‐rich medicinal plants represent valuable candidates for the development of adjunct therapies against chemotherapy‐induced renal damage [6].

*Mercurialis annua* (Euphorbiaceae), traditionally used in the Mediterranean region for the treatment of numerous illnesses. Ethnopharmacological surveys have reported its applications as a purgative, anxiolytic, and remedy for infertility, kidney ailments, and respiratory conditions [12]. Phytochemical investigations have revealed that *M. annua* is rich in bioactive compounds such as flavonoids (quercetin, rutin, and narcissin), phenolic acids, and other polyphenols, which are recognized for their potent antioxidant and anti‐inflammatory activities [13]. These pharmacological properties provide a scientific rationale for investigating its potential protective effects against oxidative stress‐mediated renal injury.

Since the phytochemical composition of medicinal plants is influenced by environmental and climatic conditions, investigating the Moroccan population of *M. annua* is of particular interest to characterize its phytochemical profile and evaluate its biological potential. Although the traditional use of *M. annua* for kidney disorders has been documented, no experimental study has comprehensively evaluated the nephroprotective activity of Moroccan *M. annua* in a cyclophosphamide‐induced nephrotoxicity model. Furthermore, the phytochemical profile of the Moroccan population of this species has not been comprehensively characterized using UHPLC‐ESI/MS, and its safety profile in relation to nephroprotective application remains insufficiently documented. Addressing these knowledge gaps, the present study combines phytochemical profiling, acute oral toxicity assessment, and evaluation of the nephroprotective efficacy of the hydroethanolic extract of *M. annua* against CP‐induced renal injury in rats. By integrating chemical characterization with biological validation, this work provides the first comprehensive evidence supporting the traditional renal use of Moroccan *M. annua* and identifies it as a potential source of nephroprotective phytochemicals (Figure 1).

**FIGURE 1:**
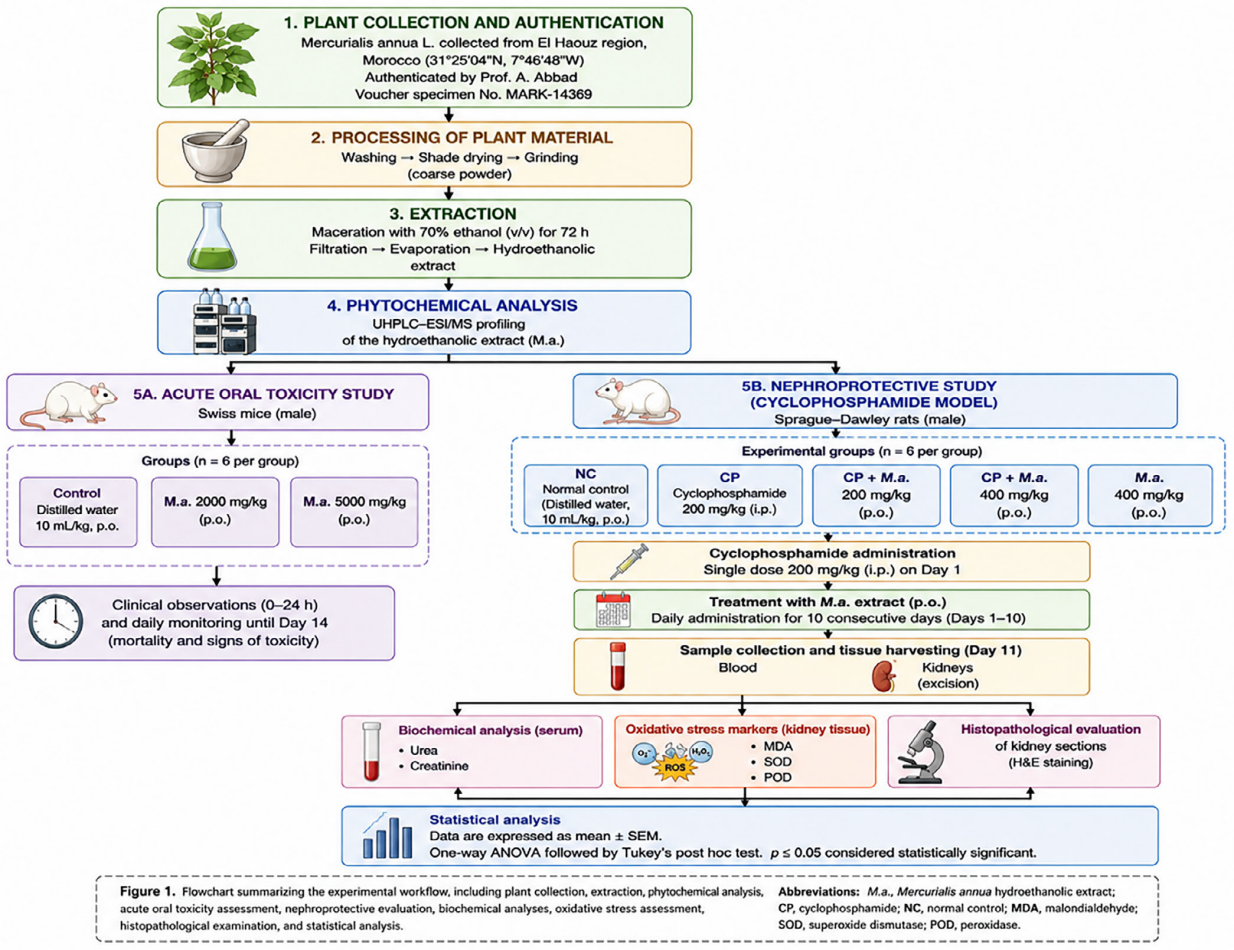
Schematic overview of the experimental workflow employed in the present study.

## 2 Materials and Methods

### 2.1 Plant Material

*M. annua* were collected from the El Haouz region in southern Morocco (31°25′04′′N, 7°46′48′′W). The plant was authenticated by Professor A. Abbad, and a voucher specimen (No. MARK‐14365) was deposited at the Herbarium of the Faculty of Sciences Semlalia, Marrakech. The collected material was washed, shade‐dried, and then coarsely ground using a grinder.

### 2.2 Extract Preparation

The hydroethanolic extract was prepared by drying and grinding 70 g of the aerial parts of *M. annua*, which were macerated in 700 mL of 70% ethanol at room temperature for 72 h with magnetic stirring for 1 h per day. The extract was then filtered and concentrated under reduced pressure using a rotary evaporator to remove ethanol. The remaining aqueous phase was subsequently freeze‐dried (lyophilized) to obtain a dry extract. The extraction yield was 18.53%.

### 2.3 Phytochemical Screening

The phytochemical screening was performed on a hydroethanolic extract obtained from dried and powdered plant material. Standard qualitative tests were conducted to identify major phytochemical groups, including the Folin–Ciocalteu test for polyphenols, aluminum chloride for flavonoids, Dragendorff's for alkaloids, Bornträger's for anthraquinones, and Liebermann–Burchard for terpenoids. Each test was performed in triplicate, and observations were based on characteristic color changes or precipitate formation [14].

### 2.4 UHPLC–ESI/MS Analysis

The hydroethanolic extract of *M. annua* was analyzed by UHPLC–ESI/MS using an electrospray ionization (ESI) source according to a previously validated method [15]. The analysis was performed on an Acquity UHPLC system (Waters, CA, USA) equipped with a quaternary pump, autosampler, and column oven. Separation was achieved on a C18 reversed‐phase column (250 × 4.6 mm, 2.6 µm; Thermo Fisher Scientific, CA, USA) using a gradient of solvent A (0.1% formic acid in water) and solvent B (methanol) at a flow rate of 0.2 mL/min. Chromatographic separation was achieved using a multistep gradient elution program of solvent B. The gradient was initiated at 5% B and linearly increased to 25% within 3 min, where it was maintained until 6 min. Solvent B was then raised to 37% at 9 min and held constant until 13 min, followed by a further increase to 54% at 18 min, which was maintained until 22 min. A steep gradient was subsequently applied to reach 95% B at 25 min and maintained until 29 min to ensure the elution of strongly retained compounds. The system was then rapidly returned to the initial conditions (5% B at 29.15 min) and equilibrated until 31 min prior to the next injection. Detection was carried out on a Xevo TQD triple quadrupole mass spectrometer (Waters, CA, USA) with a heated ESI source operating in negative mode under full scan (*m*/*z* 50–1000). The ion transfer and vaporizer temperatures were maintained at 250°C with an electrospray voltage of 3500 V.

### 2.5 Animals

Adult male Sprague–Dawley rats and Swiss mice were provided by the animal facility of the Biology Department at the Faculty of Sciences Semlalia, Cadi Ayyad University in Marrakech (Morocco). They were maintained in standard laboratory conditions (temperature controlled at 25 ± 2°C, a 12‐h light/dark cycle, and allowed unrestricted access to food and water ad libitum).

This study strictly followed ethical norms and principles to ensure the welfare of the animal subjects, including those stated in the Institutional Animal Care and Use Committee (IACUC) and in accordance with national and international regulations (EU2010/63). Ethical approval was obtained in advance from the Institutional Ethics Committee of the Faculty of Sciences, Cadi Ayyad University, Marrakech, Morocco (Protocol No. BA‐05/2025). Measures were taken to improve animal living circumstances, such as proper nourishment, temperature regulation, and social isolation.

Swiss albino mice were selected for the acute oral toxicity study in accordance with OECD guideline, which recommends mice as a standard species for acute oral toxicity testing [16]. Sprague–Dawley rats were selected for the nephroprotective study because they are a well‐established and widely used experimental model for investigating drug‐induced renal injury, including cyclophosphamide‐induced nephrotoxicity [5].

### 2.6 Toxicity Study

For the acute oral toxicity study, 18 adult male mice (30 ± 5 g) were used. The study was conducted in accordance with the principles of OECD guideline 423 [16], with minor adaptation as previously described by Benrahou et al., 2022 [17]. Adult male mice were used instead of rat to align with local ethics and previous bioassay models [18]. The animals were divided into three main groups (*n* = 6 per group) and treated using a sequential stepwise procedure (3 mice per step).

Initially, a first step of 3 mice received a single oral dose of *M. annua* extract at 2000 mg/kg, since no mortality, behavioral alteration, or signs of toxicity were observed within 48‐h, a second step of 3 mice was treated at the same dose 2000 mg/kg to confirm the safety. All mice were subsequently observed for 14 days for mortality, clinical signs of toxicity, behavioral changes, and body weight changes. No mortality or treatment‐related signs of toxicity were observed, and the animals remained in good general condition throughout the observation period. Subsequently, to verify a higher safety margin suitable for medicinal extracts, an extended limit test at 5000 mg/kg was performed in tow sequential steps of 3 mice in each under identical conditions. Control animals (*n* = 6) received distilled water orally and were monitored under the same conditions.

### 2.7 Experimental Design

The experimental design involved thirty adult male Sprague–Dawley rats weighing 250 ± 15, randomly distributed into five distinct groups.

Group 1 (control) received distilled water orally for ten consecutive days.

Group 2 (toxic control) was exposed to a single intraperitoneal dose of cyclophosphamide (200 mg/kg) on day one and received distilled water orally for 10 days.

Groups 3 and 4 were subjected to the same cyclophosphamide protocol, followed by oral administration of *M. annua* extract at doses of 200 and 400 mg/kg, respectively, once daily for 10 days.

Group 5 received only the plant extract at 400 mg/kg during the same period, without cyclophosphamide exposure.

The extract doses (200 and 400 mg/kg) were selected based on previous pharmacological studies demonstrating their biological efficacy [19]. Cyclophosphamide was administered as a single intraperitoneal dose (200 mg/kg) to establish a well‐characterized acute nephrotoxicity model. This protocol has been widely used in experimental studies because it induces reproducible oxidative stress‐mediated renal injury while maintaining acceptable survival, making it suitable for evaluating nephroprotective agents [10, 20].

After the final treatment, animals were euthanized under anesthesia with chloral hydrate. Blood of each rat was collected for serum separation by centrifugation (4000 g, 10 min). Kidneys were rapidly excised, and representative tissue samples were homogenized in cold phosphate buffer. After centrifugation at 10,000 g for 15 min at 4°C, the supernatants were used for the biochemical assessment of lipid peroxidation and antioxidant enzyme activities.

### 2.8 Body and Kidney Weight Measurement

At the end of the experimental period, the animals were weighed immediately before sacrifice to obtain the final body weight. Both kidneys were carefully excised, rinsed with ice‐cold saline to remove residual blood, blotted dry with filter paper, and weighed individually using an analytical balance. The relative kidney weight was calculated separately for each kidney by dividing its weight by the final body weight of the corresponding animal. For presentation purposes, the resulting ratio was expressed as ×10^3^. For each animal, the relative kidney weights of the right and left kidneys were then averaged. The relative kidney weight was calculated according to the following formula:

$$\mathrm{Relative}\mathrm{kidney}\mathrm{weight}=\left[\frac{\mathrm{Kidney}\mathrm{weight}\left(g\right)}{\mathrm{Final}\mathrm{body}\mathrm{weight}\left(g\right)}\right]\times 10^{3}$$

### 2.9 Kidney Function Markers

Creatinine levels (SCr) were determined using the Jaffé method at 340 nm, which involves mixing 100 µL of serum with 1 mL of alkaline picrate reagent [21].

Urea levels (BUN) were quantified using the urease enzymatic method. A reaction mixture consisting of 20 µL of serum and 1 mL of urease reagent.

Calibration curves were prepared using standard solutions for both creatinine and urea to ensure accurate quantification. All measurements were performed using a UV–vis spectrophotometer.

### 2.10 Oxidative Stress Markers

#### 2.10.1 Assay of Malondialdehyde

Lipid peroxidation, malondialdehyde (MDA), was quantified using the thiobarbituric acid reactive substance (TBARS) assay [22].

#### 2.10.2 Superoxide Dismutase Activity Assay

Photochemical reduction of tetrazolium nitro blue was measured at 560 nm in order to determine the activity of the superoxide dismutase enzyme (SOD) according to Beyer and Fridovich [23].

#### 2.10.3 Peroxidase Activity

Guaiacol was used as a hydrogen donor to test peroxidase activity at 470 nm. The reaction mixture was composed of 1.1 g/L guaiacol and 0.06% hydrogen peroxide (H_2_O_2_), both dissolved in 13.8 g/L phosphate buffer. An aliquot of 50 µL enzyme extract was added, and the total volume was adjusted to 1 mL. Peroxidase activity was calculated using a molecular extinction coefficient of 26.2 mM^−^^1^ and expressed in µmol of guaiacol oxidized per mg of protein per minute [24].

### 2.11 Histopathological Examination of Kidney Tissues

For the histopathological examination, kidneys were carefully excised and conserved in a 4% neutral buffered formalin solution for preservation over 72 h. Following fixation, the specimens underwent a dehydration process through successive ethanol baths of increasing concentrations. They were then embedded in paraffin wax at controlled temperatures (60°C–70°C). Thin slices, approximately 4 micrometers thick, were prepared with a rotary microtome. These slices were mounted on glass slides, dried in an incubator for 20 min, and subsequently stained with hematoxylin and eosin.

Kidney sections were examined by a blinded pathologist. Renal lesions, including glomerular atrophy (GD), tubular degeneration (TD), inflammatory cell infiltration (INF), and hemorrhage (HEM), were semiquantitatively scored using a four‐grade scale adapted from [Reference]: 0 = no damage; 1 = mild damage (0%–25% of the examined tissue affected); 2 = moderate damage (25%–75%); and 3 = severe damage (75%–100%). The score assigned for each criterion represented the severity and extent of the corresponding histopathological lesion [25].

### 2.12 Statistical Analysis

Data are presented as mean ± SEM. Biochemical, oxidative stress, and other quantitative data were analyzed using one‐way analysis of variance (ANOVA) followed by Tukey's multiple comparison test. Histopathological scores were analyzed using the Kruskal–Wallis test followed by Dunn's multiple comparisons test. A *p* value < 0.05 was considered statistically significant. All statistical analyses and graphical representations were performed using GraphPad Prism version 9.0 (GraphPad Software Inc., San Diego, CA, USA)

## 3 Results and Discussion

### 3.1 Phytochemical Screening and UHPLC‐ESI/MS Analysis

Phytochemical screening of the hydroethanolic extract of *M. annua* revealed many key bioactive compounds, including polyphenols, flavonoids, alkaloids, and coumarins. Moderate amounts of terpenoids and saponins were also detected. Tannins showed a slight presence, while anthraquinones were absent (Table 1). Nevertheless, variations in phytochemical composition among studies are frequently reported and may result from differences in geographical origin, environmental conditions, harvesting period, plant part used, and extraction procedures [26]. The occurrence of these secondary metabolites is particularly relevant because numerous studies have demonstrated their antioxidant, anti‐inflammatory, and cytoprotective activities, suggesting that they may contribute to the nephroprotective effects observed in the present study [27].

**TABLE 1:** Preliminary phytochemical screening of the hydroethanolic extract of *M. annua*.

Test / Reagent used	Target compound	Presence
Folin‐Ciocalteu test	Polyphenols	+
Ferric chloride (FeCl_3_)	Tannins	+
Aluminum chloride test	Flavonoids	+
Dragendorff's test	Alkaloids	+
Bornträger's test	Anthraquinones	—
NH_4_OH and UV exposure	Coumarins	+
Liebermann–Burchard test	Terpenoids	+
Foam test	Saponins	±

*Note*: (+) = present; (±) = weakly present; (–) = absent. Tests were based on characteristic color changes or precipitate formation.

In Total, 15 compounds were detected (Figure 2A,B) in negative ionization mode. In the absence of authentic standards and comprehensive MS/MS data, only 7 compounds were tentatively identified (Table 2), based on MS data and published reports.

**FIGURE 2:**
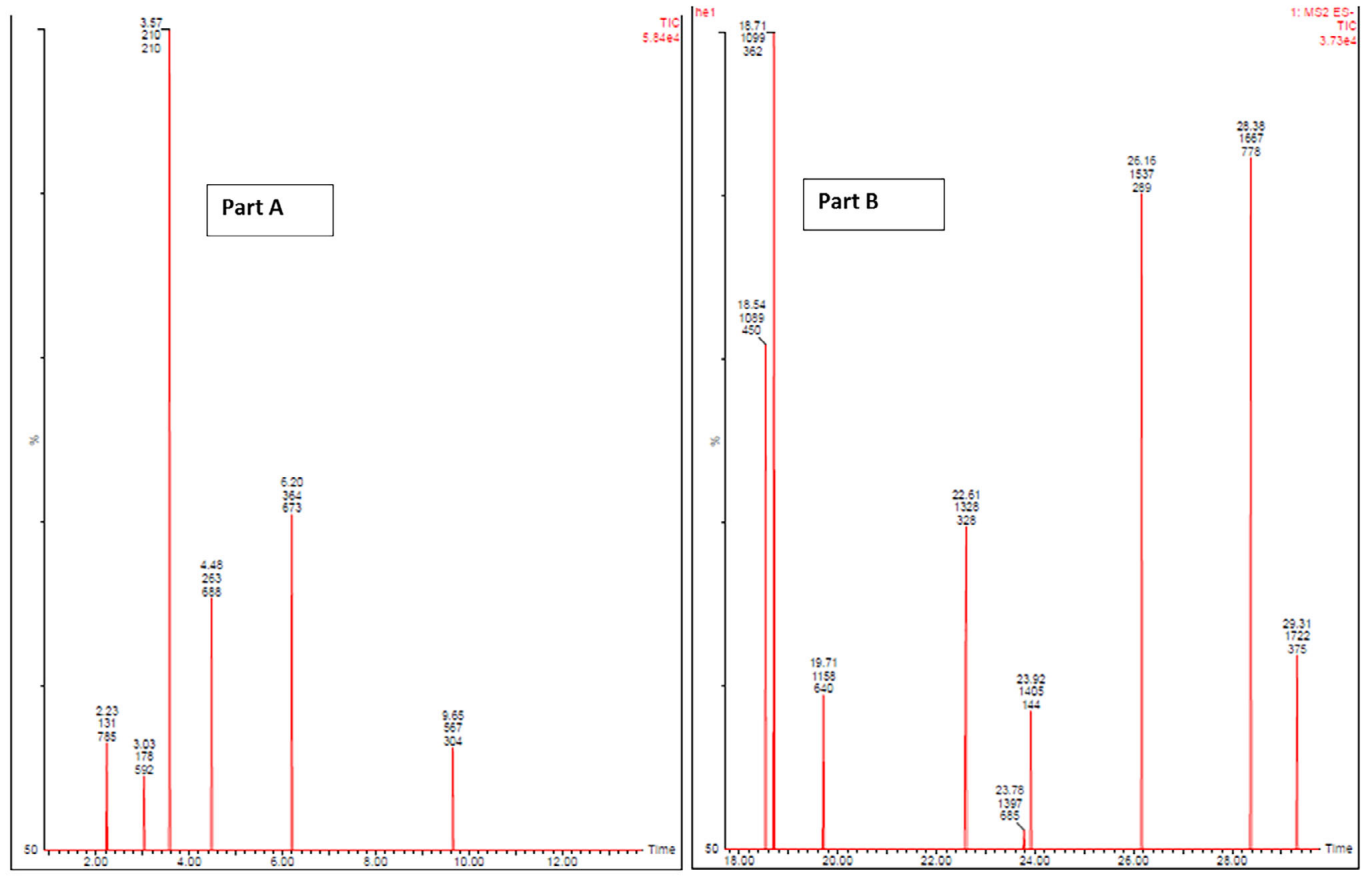
A–B: UHPLC profile of the hydroethanolic extract of *M. annua*.

**TABLE 2:** Tentative UHPLC‑ESI/MS identification of bioactive compounds in the hydroethanolic extract of *M. annua*.

No	RT	*m*/*z* (M−H)	Fragment	Proposed compound^a^	Molecular formula	Relative area (%)
1	2,23	785	301	Isorhamnetin‐3‐O‐rutinoside‐7‐O‐glucoside	C_34_H_42_O_21_	6.81
2	3,57	364 (M−H + HCOOH)	210	dihydroisorhamnetin	C_16_H_14_O_7_	18.39
3	4,48	688 (2M−H)	—	3‐(2,4‐Dihydroxyphenyl) propanoic acid hexoside	C_15_H_20_O_9_	9.64
6	9,6	303	—	Taxifolin (dihydroquercetin)	C_15_H_12_O_7_	4.25
8	18,71	362	347	Salvicinolide methyl ester	C_21_H_29_O_5_	5.66
9	22,61	327	—	Hydro‐*p*‐coumaric acid‐hexoside	C_15_H_20_O_8_	16.46
10	26,16	289	—	Epicatechin	C_15_H_14_O_6_	4.14

*Note*: Compounds were tentatively identified based on their retention time (RT), deprotonated molecular ions ([M−H]^−^), fragmentation patterns (MS/MS), molecular formula, and comparison with data reported in the literature. Relative area (%) represents the relative abundance of each detected compound and was calculated from the corresponding UHPLC‐ESI/MS chromatographic peak area.

**Abbreviations**: [M−H]^−^, deprotonated molecular ion detected in negative ionization mode; HCOOH, formic acid; *m*/*z*, mass‐to‐charge ratio; MS/MS, tandem mass spectrometry; RT, retention time.

^a^ compound tentatively identified based on UHPLC‐ESI/MS data and literature comparison.

The most abundant compound, detected at RT 16.32 min, was provisionally characterized as dihydroisorhamnetin (18.39%), an O‐methylated flavonol, observed in its dimeric form associated with formic acid (HCOOH) at *m*/*z* 364. Another prominent constituent, hydro‐p‐coumaric acid‐hexoside, a glycosylated derivative of p‐coumaric acid (16.46%, *m*/*z* 327), eluting at 22.61 min, was putatively assigned as a conjugated phenolic acid; however, MS/MS confirmation is required. At 4.48 min, 3‑(2,4‑dihydroxyphenyl) propanoic acid hexoside (9.64%) appeared as a deprotonated dimer at *m*/*z* 688 (2M−H^−^), consistent with a monomer [M−H]^−^ ≈ 343; MS/MS data are needed to confirm hexose loss (−162) and aglycone fragments. Isorhamnetin‑3‑O‑rutinoside‑7‑O‑glucoside (or its isomer isorhamnetin‑3‑O‑rutinoside‑4'‐O‑glucoside (6.81%)), eluting at 2.23 min, was tentatively identified at *m*/*z* 785. Salvicinolide methyl ester (*m*/*z* 362, fragment at *m*/*z* 347, 5.66%), detected at 18.71 min, was proposed as a diterpenoid lactone bearing a methyl ester. This compound is commonly reported in plant extracts, particularly within the Asteraceae family such as *Pulicaria crispa* and *incisa* [28]. MS/MS confirmation (e.g., CO_2_ and H_2_O neutral losses) is essential for unambiguous identification. A flavanonol (taxifolin, RT 11.25 min, *m*/*z* 303) and a flavan‐3‐ol (epicatechin, RT 9.84 min, *m*/*z* 289) were also detected, albeit in lower proportions. As mentioned above, their assignment, in the absence of authentic standards, relies solely on accurate mass matching and therefore remains putative. Several peaks (e.g., *m*/*z* 673, 495, and 778) with notable relative areas remain unassigned, suggesting the presence of novel or less‑characterized compounds. Overall, the seven tentatively identified compounds represent approximately 65% of the total chromatographic area. The unassigned peaks represent about 35% of this area, thus highlighting the partial nature of the phytochemical characterization we performed.

UHPLC‐ESI/MS analysis was undertaken to characterize its phytoconstituents, with a specific focus on phenolic compounds. Very few studies have described the phenolic profile of *M. annua* using UHPLC or LC/MS/MS, and none have focused on the Moroccan species. This analytical approach was chosen for its effectiveness in separating and detecting constituents within complex natural product mixtures, such as the hydroethanolic extract of *M. annua*. In the present study, UHPLC‐ESI/MS analysis revealed fifteen compounds, seven of which were tentatively identified based on accurate mass and, where available, fragment ions, in the absence of authentic standards and comprehensive MS/MS data.

Comparison of our findings with published reports revealed close similarities. The glycosylated flavonol identified at *m*/*z* 785, tentatively assigned as isorhamnetin‐3‐O‐rutinoside‐7‐O‐glucoside (or its isomer, isorhamnetin‐3‐O‐rutinoside‐4'‐O‐glucoside), showed an unexpected fragmentation pattern: The expected aglycone ion at *m*/*z* 315 (isorhamnetin) was not detected. Instead, a fragment ion at *m*/*z* 301 (C_15_H_9_O_7_) was observed, consistent with a quercetin‐type nucleus generated by successive losses of rhamnose+glucose (−308), glucose (−162), and a partial methoxy group (≈−14), that is [(M−H)−(308 + 162 + 14)]^−^.

The simultaneous presence of this glycosylated derivative and dihydroisorhamnetin suggests a possible metabolic or degradative relationship, with dihydroisorhamnetin potentially arising from the in vivo hydrolysis of isorhamnetin glycosides, from in‐source transformations during analysis, or through potential hydrogenation of isorhamnetin. This association highlights the structural relationship between glycosylated flavonols and their reduced analogues in the extract.

The detection of isorhamnetin‐3‐O‐rutinoside‐7‐O‐glucoside (or isomer) corroborates [29], while the putative assignment of epicatechin is consistent with the Tunisian findings of [30]. In contrast, taxifolin, a dihydroquercetin, together with salvicinolide methyl ester, dihydroisorhamnetin, and hydro‐pCoA‐hexoside, although commonly found in other plants, are documented here for the first time in *M. annua*. Overall, the comparison with published reports in the literature, according to geographic origin, harvest period, and extraction methods, not only underscores a certain variability in the phytochemical profile of *M. annua* but also highlights some noteworthy similarities with previous findings. For instance: phaselic acid in German aqueous extracts [31], narcissin, rutin, epicatechin, epigallocatechin and gallocatechin in Tunisian hydromethanolic extracts [30], classical flavonoids such as kaempferol, quercetin, isorhamnetin and rutin in Jordanian ethanolic extracts [13], rutin, narcissin, and isorhamnetin‐3‐O‐rutinoside‐7‐O‐glucoside together with its isomer isorhamnetin‐3‐O‐rutinoside‐4'‐O‐glucoside in a Southern Italian (Naples) methanolic extract [29], and a broader diversity in Moroccan hydroethanolic extract. These differences highlight the combined influence of the variability factors mentioned above, and emphasize the originality of our contribution to the phytochemical characterization of *M. annua*.

The bioactive compounds listed in Table 2 are well documented for their antioxidant and anti‐inflammatory properties, which can protect renal tissues against oxidative and inflammatory damage [32, 33, 34, 35]. However, it should be emphasized that the hydroethanolic extract examined here represents a complex mixture of phenolic and terpenoid compounds, such as those presented in Table 2, but also other secondary metabolites whose presence was evidenced in Table 1 (tannins, alkaloids, coumarins, and saponins). In fact, in addition to the compounds already reported, the exclusive occurrence of alkaloids such as pyridine‐3‐carbonitrile and nicotinamide was demonstrated in the CH_2_Cl_2_ extract obtained from the aerial parts of *M. annua* [36].

Following this partial phytochemical characterization, it should be noted that no authentic standards were available for confirming the retention time of the compounds listed in Table 2, including the major constituents epicatechin and taxifolin; therefore, all assignments should be regarded as putative annotations based on accurate mass matching, pending confirmation by coinjection with reference standards and/or MS/MS fragmentation analysis. Fractionation by column chromatography followed by characterization using standard spectroscopic techniques (IR, UV, HRMS, and NMR (^1^H and ^13^C)) would allow the unambiguous structural elucidation of the compounds provisionally identified here.

### 3.2 Acute Toxicity

The oral administration of *M. annua* extract at doses of 2000 and 5000 mg/kg was evaluated for acute toxicity. No mortality or visible signs of toxicity were recorded in any of the six mice in the test groups at either dose level. Additionally, no significant changes in body weight were when comparing treated groups to control throughout the 14‐day observation period. This finding aligns with previous studies reporting the nontoxic nature of *M. annua* extracts at high doses [37]. The unpleasant side effects observed after consuming specific plants or their derivatives are caused by the presence of dangerous secondary metabolites [38]. Interestingly, previous studies have reported that *M. annua* extracts possess a certain degree of selectivity toward cancer cells, as reflected by their higher cytotoxicity against malignant cell lines compared to noncancerous HEK‐293 cells [39]. Although the absence of mortality, clinical signs of toxicity, and body weight changes supports the favorable acute safety profile of the extract, these parameters alone are not sufficient to establish its overall toxicological safety. Additional investigations, including hematological, biochemical and histopathological studies, are required to comprehensively assess its safety. Nevertheless, acute toxicity assessment alone does not provide comprehensive information regarding long‐term safety. Therefore, subchronic and chronic toxicity studies remain necessary before considering future therapeutic applications.

### 3.3 Effects of *M. Annua* on Body, Kidney, and Relative Kidney Weight

Cyclophosphamide administration induced a progressive loss of body weight throughout the experimental period. As shown in Table 3, CP‐treated (200 mg/kg) rats exhibited body weight reductions of 8.91% on Day 1, 20.08% on Day 5, and 25.31% on Day 10 compared with the initial body weight (*p* < 0.001). Treatment with *M. annua* attenuated this weight loss. At 200 mg/kg, body weight loss was reduced to 5.67%, 14.13%, and 8.13% on Days 1, 5, and 10, respectively. The 400 mg/kg dose provided greater protection, limiting body weight changes to 3.84% on Day 1, 3.04% on Day 5, and only 0.81% on Day 10, indicating an almost complete prevention of CP‐induced body weight loss by the end of the treatment period.

**TABLE 3:** Effect of *M. annua* and cyclophosphamide on body weight change.

	Body change %				
Day	Control	CP 200 mg/Kg	CP + M.a 200 mg/Kg	CP + M.a 400 mg/Kg	M.a 400 mg/Kg
1	0.09 ± 0.13	−8.91 ± 0.59 ^###^	−5.67 ± 0.64 ***	−3.84 ± 0.46 ^#^ ***	1.23 ± 0.36 ***
5	0.30 ± 0.78	−20.08 ± 1.57 ^###^	−14.13 ± 1.38 *** ^###^	−3.04 ± 1.26 ^#^ ***	1.77 ± 0.66 ***
10	2.02 ± 0.67	−25.31 ± 1.34 ^###^	−8.13 ± 0.92 *** ^###^	−0.81 ± 0.72 ***	3.10 ± 0.66 ***

*Note*: Body weight changes are expressed as percentage (%) relative to the initial body weight (Day 0) and presented as mean ± SEM (*n* = 6). Statistical analysis was performed using one‐way ANOVA followed by Tukey's multiple comparisons test. Comparisons versus the Control group are indicated by # (# *p* < 0.05, ## *p* < 0.01, ### *p* < 0.001), whereas comparisons versus the CP group are indicated by * (* *p* < 0.05, ** *p* < 0.01, *** *p* < 0.001).

**Abbreviations**: CP, cyclophosphamide (200 mg/kg); M. a, *Mercurialis annua* hydroethanolic extract (200 or 400 mg/kg); SEM, standard error of the mean.

As presented in Table 4, cyclophosphamide administration significantly increased both absolute and relative kidney weights compared with the control group (*p* < 0.001). Treatment with *M. annua* at both 200 and 400 mg/kg significantly reduced these increases compared with the CP group (*p* < 0.001). However, kidney weights in the 200 mg/kg group remained significantly higher than those of the control animals, whereas no significant difference was observed between the 400 mg/kg‐treated group and the control group, indicating an almost complete normalization of kidney weight at the higher dose.

**TABLE 4:** Effect of *M. annua* and cyclophosphamide on kidney weight and relative kidney weight.

	Kidney weight (mg)	Relative kidney weight *10^3^
**Control**	817.40 ± 24.73	2.47 ± 0.12
**CP 200 mg/Kg**	1005.80 ± 15.63 ^###^	4.21 ± 0.16 ^###^
**CP + M.a 200 mg/Kg**	942.80 ± 17.57 *** ^###^	2.85 ± 0.07 ***
**CP + M.a 400 mg/Kg**	823.20 ± 7.62 ***	2.83 ± 0.08 ***
**M.a 400 mg/Kg**	811.00 ± 10.64 ***	2.90 ± 0.04 ***

*Note*: Kidney weight and relative kidney weight are presented as mean ± SEM (*n* = 6). Relative kidney weight was calculated as (kidney weight (g)/body weight (g)) × 10^3^. Statistical analysis was performed using one‐way ANOVA followed by Tukey's multiple comparisons test. # Indicates comparisons with the Control group (### *p* < 0.001), whereas * indicates comparisons with the CP group (*** *p* < 0.001).

**Abbreviations**: CP, cyclophosphamide (200 mg/kg); M. a, *Mercurialis annua* hydroethanolic extract (200 or 400 mg/kg); SEM, standard error of the mean.

Cyclophosphamide administration induced a significant reduction in body weight together with an increase in relative kidney weight. Although an elevated relative kidney weight is commonly associated with renal edema and tissue injury, it should be interpreted with caution because the marked reduction in body weight may have partially contributed to this increase [40]. Nevertheless, the concurrent deterioration in renal function biomarkers and histopathological alterations support that these changes reflected renal damage rather than a consequence of body weight loss alone [10]. In the present study, treatment with *M. annua* attenuated these alterations, suggesting that the extract alleviated systemic toxicity while preserving renal tissue integrity. Similar improvements in body weight and relative kidney weight have been reported for other phenolic‐rich medicinal plants evaluated against cyclophosphamide‐induced nephrotoxicity, supporting the protective role of natural antioxidants in limiting renal tissue damage [41, 42].

### 3.4 Effects of *M. annua* and Cyclophosphamide on Renal Biomarkers

According to Figure 3, the CP‐treated group showed considerably higher plasma levels of creatinine and urea (*p* < 0.001) compared to the normal group. In opposition, the treatment with 200 and 400 mg/kg of *M. annua* extract significantly reduced this rise in both parameters (*p* < 0.001). Moreover, there was no apparent change in the plasma concentration of urea and creatinine between the groups who received treatment with *M. annua* and the control group. The administration of cyclophosphamide (CP) led to a significant rise in creatinine and urea concentration in serum, indicating impaired renal function.

**FIGURE 3:**
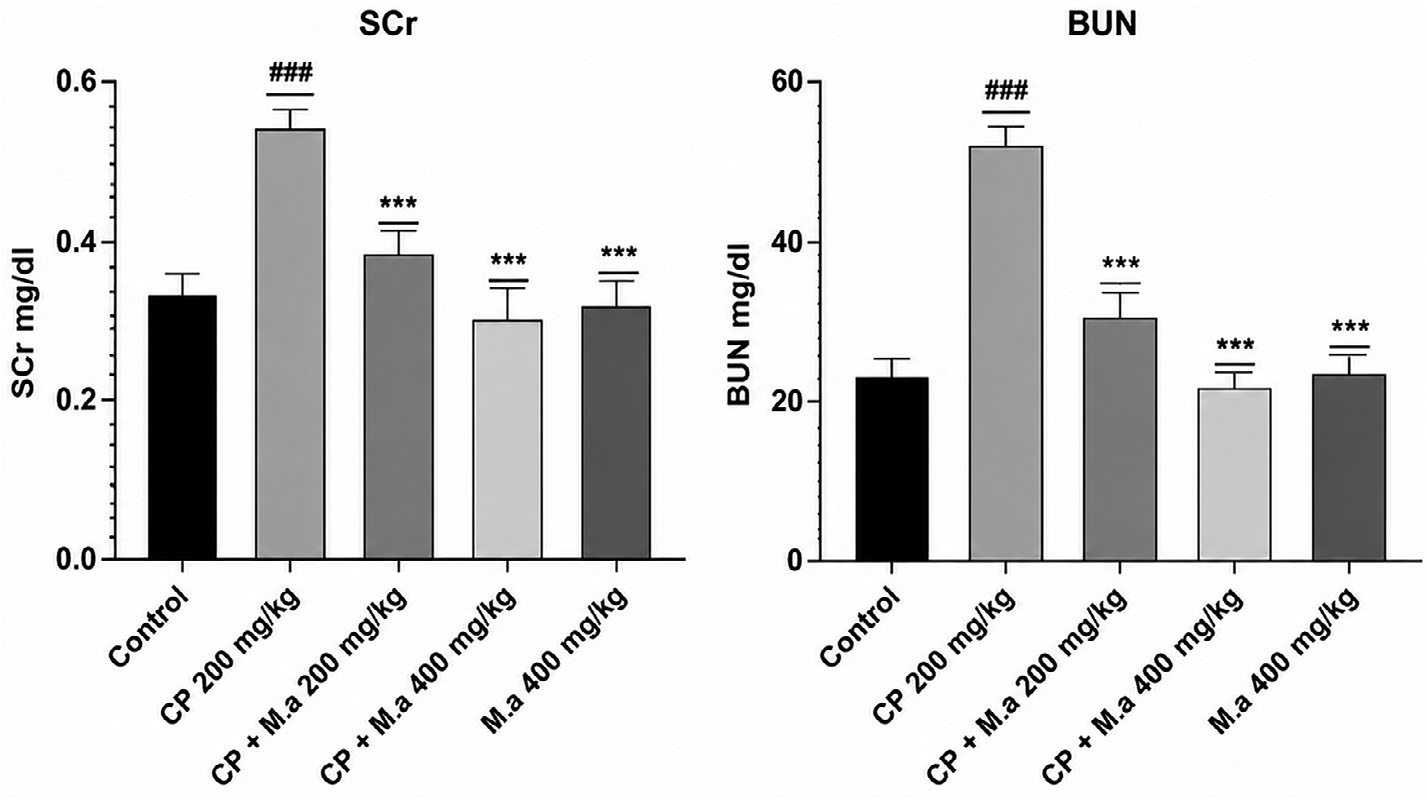
Effect of *M. annua* hydroethanolic extract on serum creatinine (SCr) and blood urea nitrogen (BUN) levels in cyclophosphamide‐induced nephrotoxicity in rats. *Note*: Serum creatinine (SCr) and blood urea nitrogen (BUN) were measured to evaluate renal function in rats following cyclophosphamide‐induced nephrotoxicity and treatment with the hydroethanolic extract of *M. annua*. Data are expressed as the mean ± SEM (*n* = 6). Statistical analysis was performed using one‐way ANOVA followed by Tukey's post hoc test. ***p < 0.001 indicate significant differences compared with the cyclophosphamide (CP) ###*p* < 0.001 indicate significant differences compared with the Control group. Abbreviations: BUN, blood urea nitrogen (mg/dL); CP, cyclophosphamide; M.a., *Mercurialis annua* hydroethanolic extract; SCr, serum creatinine (mg/dL); SEM, standard error of the mean.

The nephrotoxic effects of cyclophosphamide were further confirmed by the elevation of serum creatinine and urea, classical biomarkers of renal dysfunction. These biomarkers generally help to evaluate renal function, particularly glomerular filtration rate (GFR), and their elevation is a hallmark of nephrotoxicity [42]. This rise can be attributed to structural and functional damage in the nephrons, primarily due to oxidative and inflammatory stress induced by cyclophosphamide. In contrast, rats treated with *M. annua* extract showed a marked decrease in creatinine and urea concentration (*p* < 0.001), suggesting an improvement in renal function. These results indicate that *M. annua* may help preserve glomerular filtration and counteract CP‐induced nephrotoxicity. Moreover, CP is widely used as a chemotherapeutic and immunosuppressive, although its therapeutic efficacy is frequently curtailed owing to its nephrotoxicity and hepatotoxicity, which is reflected by elevation of creatinine, BUN, uric acid level, and liver enzymes such as ALT, AST, and GGT [5]. The restoration of renal biomarkers suggests that the extract effectively mitigated functional impairment associated with oxidative tissue damage.

### 3.5 Effects of *M. annua* and Cyclophosphamide on Oxidative Stress Markers in the Kidney Tissues

As depicted in Figure 4, the administration of cyclophosphamide led to a pronounced disturbance in the oxidative balance of renal tissues. This was evidenced by a significant decrease in enzymatic activities of superoxide dismutase (SOD) and peroxidase (POD) (*p* < 0.001), two major antioxidant enzymes involved in cellular defense against oxidative stress. Simultaneously, a marked elevation in malondialdehyde (MDA) levels was observed (*p* < 0.001). Interestingly, rats treated with 200 mg/kg of *M. annua* exhibited a substantial improvement in antioxidant status, reflected by a rise in SOD levels (*p* < 0.05) and a notable reduction in MDA concentrations (*p* < 0.01). Even more remarkable, the group receiving *M. annua* extract at 400 mg/kg presented a strong enhancement in both SOD and POD activities, along with a substantial decline in lipid peroxidation levels (MDA) (*p* < 0.001).

**FIGURE 4:**
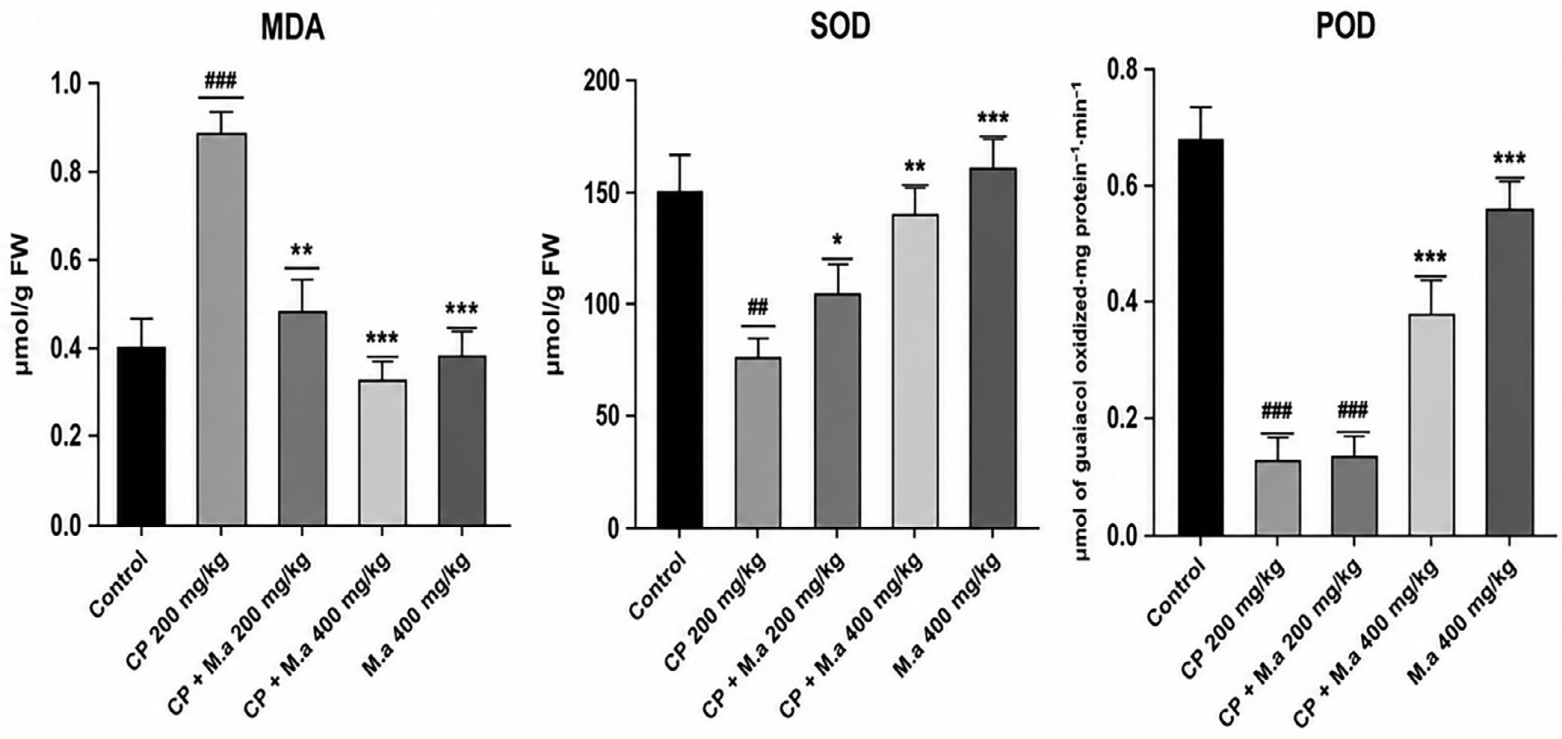
Effect of *M. annua* hydroethanolic extract on renal oxidative stress biomarkers in cyclophosphamide‐induced nephrotoxicity in rats. *Note*: The levels of malondialdehyde (MDA), superoxide dismutase (SOD), and peroxidase (POD) were determined in kidney homogenates following treatment with *M. annua* hydroethanolic extract. Data are presented as the mean ± SEM (*n* = 6). Statistical analysis was performed using one‐way ANOVA followed by Tukey's post hoc test. * *p* < 0.05, **p < 0.01, and ***p < 0.001 indicate significant differences compared with the cyclophosphamide (CP) group; #*p* < 0.05, ##*p* < 0.01, and ###*p* < 0.001 indicate significant differences compared with the Control group. **Abbreviations**: CP, cyclophosphamide; FW, fresh weight; M.a, *Mercurialis annua* hydroethanolic extract; MDA, malondialdehyde (µmol/g FW); POD, peroxidase (µmol of guaiacol oxidized·mg protein^−^^1^·min^−^^1^); SEM, standard error of the mean; SOD, superoxide dismutase (µmol/g FW).

Oxidative damage is commonly assessed by measuring malondialdehyde (MDA) [43]. In this experience, MDA levels were expressively increased in the group treated with CP (*p* < 0.001), reflecting enhanced ROS production and membrane lipid damage [42]. Notably, the treatment with *M. annua* significantly reduced MDA concentrations, indicating a protective effect against membrane damage caused by CP. Oxidative stress caused by CP administration resulted in a substantial decrease of the antioxidant enzyme activity, as well as superoxide dismutase (SOD) and peroxidase (POD). The suppression of these enzymes indicates the weakness of the antioxidant system and increased susceptibility to nephrotoxicity, since these enzymes are essential in counterbalancing reactive oxygen species (ROS) and maintaining cellular redox equilibrium [8]. However, *M. annua* treatment significantly restored the activities of both SOD and POD (*p* < 0.05, *p* < 0.01). This enhancement of antioxidant enzyme activities underscores the *M. annua*’s ability to activate endogenous defense systems and reduce oxidative damage in renal tissues and is suggested as a key mechanism underlying its protective effects. The increased activity of SOD contributes to the scavenging of superoxide anions (O_2_^−^) generated by CP. Meanwhile, peroxidase (POD) has a vital role in neutralizing hydrogen peroxide (H_2_O_2_) by converting it into water (H_2_O) and molecular oxygen (O), thereby avoiding the accumulation of cytotoxic reactive oxygen species [43]. These findings are consistent with previous studies showing that *Allium jesdianum, Curcuma caesia*, and *Medicago sativa* attenuate cyclophosphamide‐induced oxidative stress by restoring endogenous antioxidant enzymes and reducing lipid peroxidation, thereby protecting renal tissue against cyclophosphamide‐induced injury [42, 44, 45].

### 3.6 Effect of *M. Annua* and Cyclophosphamide on Renal Histology

Histologically (Figure 5), cyclophosphamide (CP) administration induced significant renal damage, as evidenced by epithelial cell degeneration, leukocyte infiltration, glomerular degeneration, enlargement of Bowman's capsule, and thickening of its basement membrane (b), in comparison with the normal renal architecture observed in the control group (a). These findings clearly indicate CP‐induced nephrotoxicity. However, coadministration of *M. annua* extract with CP (c and d) resulted in a marked attenuation of renal damage, as demonstrated by reduced glomerular degradation, decreased inflammatory cell infiltration, and a more preserved renal tissue structure compared to the CP‐treated group alone. Furthermore, normal histological features were maintained in both the extract‐only group (e) and the normal control group (a). Histological examination corroborated the biochemical findings. Kidney sections from CP‐treated rats exhibited severe pathological changes consistent with those previously reported, including glomerular damage, tubular necrosis, and inflammatory infiltration [42, 46]. In contrast, rats treated with *M. annua* displayed substantial histological improvement, with preserved renal architecture and reduced signs of glomerular and tubular degeneration. These histopathological improvements reinforce the biochemical evidence of renal protection and confirm the tissue‐level efficacy of *M. annua* against CP‐induced nephrotoxicity. Similar histopathological protection has also been reported for *Croton macrostachyus* (Euphorbiaceae), which attenuated cyclophosphamide‐induced renal damage, suggesting that nephroprotective properties may be shared among members of this plant family [10].

**FIGURE 5:**
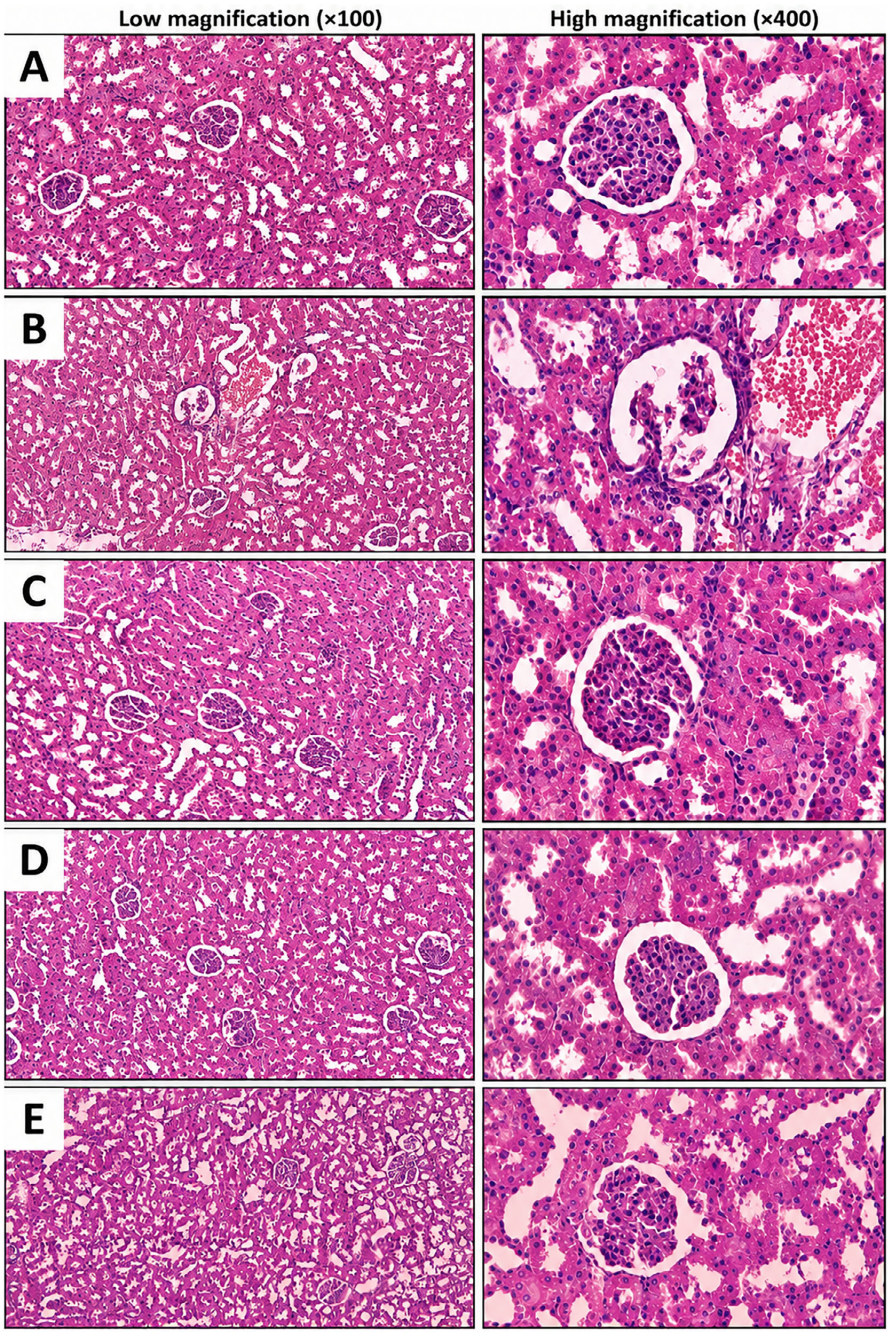
Illustrative magnification of renal sections (H&E; × 100 and 400 magnifications). The renal sections from the CP rats show a loss of the normal renal architecture, with marked infiltration of periglomerular interstitial inflammatory cells (B). The treatment of animals with *M. annua* extract revealed a better preservation similar to the control group.**Abbreviations**: A: normal control; B: cyclophosphamide at dose of 200 mg/kg; C&D cyclophosphamide plus *M. annua*, at doses of, 200, and 400 mg/kg respectively; E: only *M. annua* at dose of 400 mg/kg.

Semiquantitative histopathological scoring (Table 5) further confirmed the protective effect of *M. annua* against CP‐induced renal injury. Compared with the control group, CP administration significantly increased the scores for glomerular atrophy (2.83 ± 0.17, *p* < 0.01), tubular dilation (2.50 ± 0.22, *p* < 0.05), inflammatory cell infiltration (2.50 ± 0.22, *p* < 0.01), and hemorrhage (2.67 ± 0.21, *p* < 0.01). Treatment with *M. annua* at 200 mg/kg partially reduced these histopathological alterations, with a significant decrease observed only for hemorrhage compared with the CP group (*p* < 0.05). In contrast, treatment with 400 mg/kg significantly decreased the scores of all evaluated parameters, including glomerular degeneration, tubular dilation, inflammatory cell infiltration, and hemorrhage (*p* < 0.05 vs. CP), restoring values close to those of the control group. Administration of *M. annua* alone (400 mg/kg) did not induce any remarkable histopathological alterations, with scores comparable to those of the control group.

**TABLE 5:** Semiquantitative histopathological scoring of renal lesions in cyclophosphamide‐induced nephrotoxicity.

	GA	TD	INF	HEM
**Control**	0.5 ± 0.34	0.50 ± 0.22	0.33 ± 0.21	0.17 ± 0.17
**CP 200 mg/Kg**	2.83 ± 0.17 **	2.50 ± 0.22 *	2.5 ± 0.22 **	2.67 ± 0.21 **
**CP + M.a 200 mg/Kg**	2.00 ± 0.26	1.17 ± 0.31	1.50 ± 0.22	2.00 ± 0.26 *
**CP + M.a 400 mg/Kg**	0.83 ± 0.31 ^#^	0.67 ± 0.33 ^#^	0.67 ± 0.21 ^#^	0.5 ± 0.22 ^#^
**M.a 400 mg/Kg**	0.67± 0.21	0.33 ± 0.21	0.50 ± 0.22	0.5 ± 0.22

*Note*: Values are expressed as mean ± SEM (*n* = 6). Renal histopathological lesions were semiquantitatively scored using a four‐grade scale: 0 = no damage; 1 = mild damage (0%–25%); 2 = moderate damage (25%–75%); and 3 = severe damage (75%–100%). Statistical analysis was performed using the Kruskal–Wallis test followed by Dunn's multiple comparisons test. *p < 0.05 and **p < 0.01 versus the Control group; #*p* < 0.05 versus the CP group.

**Abbreviations**: CP, cyclophosphamide; GA, glomerular atrophy; HEM, hemorrhage; INF, inflammatory cell infiltration; M.a, *Mercurialis annua*; SEM, standard error of the mean; TD, tubular degeneration.

Histopathological examination provided structural confirmation of the biochemical findings. Cyclophosphamide administration induced marked renal lesions, including glomerular degeneration, tubular degeneration, inflammatory cell infiltration, and hemorrhage, consistent with the well‐documented renal toxicity associated with its reactive metabolite, acrolein [46]. These structural alterations reflect severe oxidative and inflammatory injury within the renal parenchyma and explain the concomitant impairment of renal function observed in CP‐treated animals [42].In contrast, treatment with *M. annua* markedly preserved renal architecture and significantly reduced the severity of these histopathological alterations. The improvement in the semi‐quantitative histopathological score was consistent with the restoration of renal function biomarkers and oxidative stress parameters, providing complementary evidence for the nephroprotective efficacy of the extract. Similar histopathological improvements have been reported for antioxidant‐rich medicinal plants in experimental models of cyclophosphamide‐induced nephrotoxicity [20, 45]. Collectively, these findings indicate that the biochemical improvements observed following *M. annua* treatment were accompanied by structural protection of renal tissue rather than merely functional changes. Although the precise molecular mechanisms were not directly investigated in the present study, the significant reduction in renal MDA levels, restoration of SOD and POD activities, improvement in renal function biomarkers, and attenuation of histopathological alterations strongly suggest that the nephroprotective effect of *M. annua* is primarily associated with its antioxidant activity. These findings indicate that the extract effectively mitigated cyclophosphamide‐induced oxidative stress and preserved both renal structure and function. Such protective effects are likely attributable to the collective contribution of the phenolic and flavonoid constituents tentatively identified by UHPLC‐ESI/MS, including isorhamnetin derivatives, taxifolin, and epicatechin, which are recognized for their potent antioxidant properties and their ability to scavenge reactive oxygen species while reinforcing endogenous antioxidant defenses [33, 34].

Although molecular signaling pathways were not examined in this study, previous investigations have shown that these phytochemicals can modulate key regulators of oxidative stress and inflammation, notably through activation of the Nrf2/ARE pathway and suppression of NF‐κB signaling, thereby reducing oxidative injury, inflammatory responses, and apoptosis in renal tissues [6]. Therefore, the improvement observed in oxidative stress biomarkers and renal histology in the present study may be explained, at least in part, by these complementary mechanisms. Nevertheless, these molecular pathways remain hypothetical in the context of the present work and should be confirmed by targeted mechanistic studies.

Although the antioxidant activity of *M. annua* appears to contribute to its nephroprotective effects, its concomitant administration with cyclophosphamide deserves careful consideration. Cyclophosphamide exerts its antitumor activity mainly through its active metabolite phosphoramide mustard, which induces DNA cross‐linking in rapidly dividing tumor cells, whereas acrolein is primarily responsible for oxidative injury in normal tissues [4]. Consequently, attenuation of oxidative stress may preferentially protect healthy organs without necessarily interfering with the principal mechanism underlying cyclophosphamide's antineoplastic activity. However, because reactive oxygen species also contribute to cyclophosphamide‐induced cytotoxicity, the possibility that potent antioxidants could partially attenuate this component of the antitumor response cannot be completely excluded [47]. This consideration highlights the importance of carefully evaluating the compatibility of antioxidant‐based adjuvant therapies with conventional chemotherapy.

### 3.7 Study Limitations

The present study has several limitations that should be acknowledged. First, Although the UHPLC–ESI/MS analysis provided a comprehensive qualitative characterization of the phytochemical profile of the Moroccan *M. annua* extract, the extract was not chemically standardized through the quantitative determination of marker compounds or the measurement of total phenolic and total flavonoid contents. Second, the nephroprotective mechanisms were inferred from biochemical, oxidative stress, and histopathological findings, but no direct molecular investigations were performed to evaluate signaling pathways associated with oxidative stress, inflammation, or apoptosis, such as the Nrf2/HO‐1, NF‐κB, or caspase pathways. In addition, renal injury was assessed using conventional biomarkers (serum creatinine and blood urea nitrogen), whereas more sensitive biomarkers of early kidney injury, including KIM‐1, NGAL, and cystatin C, were not evaluated. Finally, because this study was conducted in healthy rats, it did not address the potential impact of the antioxidant properties of *M. annua* on the antitumor efficacy of cyclophosphamide, which remains an important translational consideration.

### 3.8 Future Scope

Future studies should confirm the phytochemical profile of *M. annua* through comprehensive MS/MS characterization and the use of authentic reference standards, followed by the isolation and biological evaluation of the major bioactive constituents. Such analyses would improve batch‐to‐batch consistency, facilitate quality control, and support the future pharmacological and translational development of *M. annua*. Additional investigations are also warranted to elucidate the molecular mechanisms underlying its nephroprotective activity by examining antioxidant, inflammatory, and apoptotic signaling pathways. The inclusion of sensitive renal injury biomarkers, together with pharmacokinetic and long‐term toxicity studies, would further strengthen the preclinical evaluation of the extract. Importantly, studies using tumor‐bearing animal models and different treatment schedules should be performed to determine whether *M. annua* can provide renal protection without compromising the antitumor efficacy of cyclophosphamide. Such investigations will be essential for assessing the translational potential of this medicinal plant as a safe adjuvant therapy during cyclophosphamide‐based chemotherapy.

## 4 Conclusions

The present study demonstrates that the hydroethanolic extract of *M. annua* exerts a pronounced nephroprotective effect in a dose‐dependent manner. The extract significantly attenuated cyclophosphamide‐induced renal injury, as reflected by improvements in body weight, renal biochemical markers, and oxidative stress parameters. This renoprotective activity is likely associated with the presence of phenolic compounds, particularly flavonoids such as isorhamnetin derivatives, taxifolin, epicatechin, and hydro‐p‐coumaric acid, which are well known for their antioxidant and cytoprotective properties.

Nevertheless, further studies are warranted to isolate and characterize the individual bioactive constituents responsible for these effects and to clarify their underlying molecular mechanisms. Such investigations would provide deeper insight into the therapeutic potential of *M. annua* and support its possible development as a nephroprotective agent.

## Author Contributions

**Abdeljalil Elmahdaoui**: investigation, conceptualization, writing – original draft, methodology, data curation. **Manal Zefzoufi**: investigation, methodology, writing – review and editing. **Lansine Diakité**: resources, investigation, visualization. **Mohammed Haida**: data curation, validation, formal analysis, resources. **Rabiaa Fdil**: investigation, resources, software, writing – original draft. **Aziz Auhmani**: validation, supervision, writing – review and editing, resources. **Stefania Garzoli**: writing – review and editing, supervision. **Zahra Sokar**: conceptualization, methodology, writing – review and editing, supervision, project administration.

## Funding

This research did not receive any specific grant from funding agencies in the public, commercial, or not‐for‐profit sectors.

## Conflicts of Interest

The authors declare no conflicts of interest.

## Declaration of Generative AI in Scientific Writing

QuillBot software was used solely for language correction and improvement during the writing and editing of this manuscript, without any impact on data collection or analysis.

## Data Availability

The data that support the findings of this study are available from the corresponding author upon reasonable request.
